# Rectal Injury Caused by a Water-Filled Condom: A Case Report

**DOI:** 10.7759/cureus.93236

**Published:** 2025-09-25

**Authors:** Yusuke Tanino, Hiroshi Homma, Yuta Ishigami, Naoki Motohashi, Masaru Hirayama

**Affiliations:** 1 Department of Emergency and Critical Care Medicine, Tokyo Medical University, Tokyo, JPN

**Keywords:** abdominal exercises, acute care surgery and trauma, condom, emergent laparotomy, rectal foreign bodies

## Abstract

Regarding rectal foreign bodies with gastrointestinal perforation, most objects are hard, and perforation by a condom has not been reported.

We describe the case of a 48-year-old man who inserted a water-filled condom into his rectum and performed abdominal crunch exercises for the purpose of sexual arousal. After several repetitions, the condom ruptured. Physical examination revealed signs of peritoneal irritation, and computed tomography confirmed the presence of free air and perirectal fluid accumulation. The patient was diagnosed with acute generalized peritonitis, and emergent laparotomy revealed a full-thickness rectal injury of approximately 10 cm. Simple closure of the damaged bowel was performed, and a proximal diverting colostomy was created. Postoperatively, the patient developed a surgical site infection and was discharged on hospital day 49 following an extended recovery period. The colostomy was subsequently closed without complications.

This case highlights that even a condom filled with water can cause upper rectal injury under increased abdominal pressure, such as abdominal exercises.

## Introduction

Rectal foreign bodies represent a challenging clinical problem that has been increasingly reported worldwide. Patients may present with a wide variety of inserted objects, ranging from sexual devices to household items, and the clinical presentation can vary from mild discomfort to life‑threatening perforation and sepsis. A recent systematic review and meta‑analysis summarized hundreds of cases and highlighted both diagnostic and therapeutic challenges, emphasizing the need for individualized management strategies [1].

In Japan, Takagaki et al. analyzed 140 cases reported in the domestic literature and described both the diversity of inserted objects and the technical difficulties associated with transanal extraction [2]. Although most rectal foreign bodies are hard objects such as bottles, glass, or sex toys, which can directly injure the mucosa and cause perforation, unusual or fragile objects may also lead to significant complications. The removal of such objects can be technically demanding, and complications such as mucosal laceration, bleeding, and perforation are not uncommon [3].

Here, we report a rare case of rectal injury caused by a water‑filled condom. To our knowledge, this specific mechanism of injury has not been previously documented in the literature. By presenting this case, we aim to highlight the potential risks associated with unconventional foreign bodies, discuss the diagnostic and therapeutic considerations, and contribute to the growing body of literature on rectal trauma.

## Case presentation

A 48-year-old man with no documented medical history engaged in abdominal exercises (crunches) with a condom inserted into his anus to achieve sexual arousal. The water-filled condom was covered with two additional condoms, resulting in triple layers; however, it ruptured inside the rectum after the completion of a series of crunches, resulting in the gradual onset of abdominal pain. Three hours later, emergency medical services (EMS) were contacted. The EMS team observed peritoneal irritation throughout the lower abdomen, so the patient was subsequently transported to our emergency center.

The patient was 175 cm in height and weighed 60 kg. His vital signs upon arrival were as follows: Glasgow coma scale, E4V5M6; respiratory rate, 24 breaths/min; oxygen saturation, 98% under oxygen mask at 6 L/min; heart rate, 84 beats/min; blood pressure, 118/68 mmHg; and body temperature, 38.9℃. His laboratory results were as follows: white blood cell count, 13,200/μL; hemoglobin, 13.2 g/dL; hematocrit, 38.8%; platelet count, 214,000/μL; aspartate aminotransferase, 20 U/L; alanine aminotransferase, 29 U/L; creatinine, 0.95 mg/dL; C-reactive protein, 7.0 mg/dL; international normalized ratio of prothrombin time, 1.27 (Table 1).

**Table 1:** Complete blood count on admission. WBC: white blood cell; Hb: hemoglobin; Hct: hematocrit; PLT: platelet; AST: aspartate aminotransferase; ALT: alanine aminotransferase; Cr: creatinine; CRP: C-reactive protein; INR: international normalized ratio

	Results	Unit	Reference Range
WBC	13.2	×10^3^/μL	3.3-8.6
Hb	13.2	g/dL	13.7-16.8
Hct	38.8	%	40.7-50.1
PLT	214	×10^3^/μL	140.0-340.0
AST	20	IU/L	8-38
ALT	29	IU/L	4-44
Cr	0.95	mg/dL	0.60-1.10
CRP	7	mg/dL	<0.3
INR	1.27		

**Figure 1:**
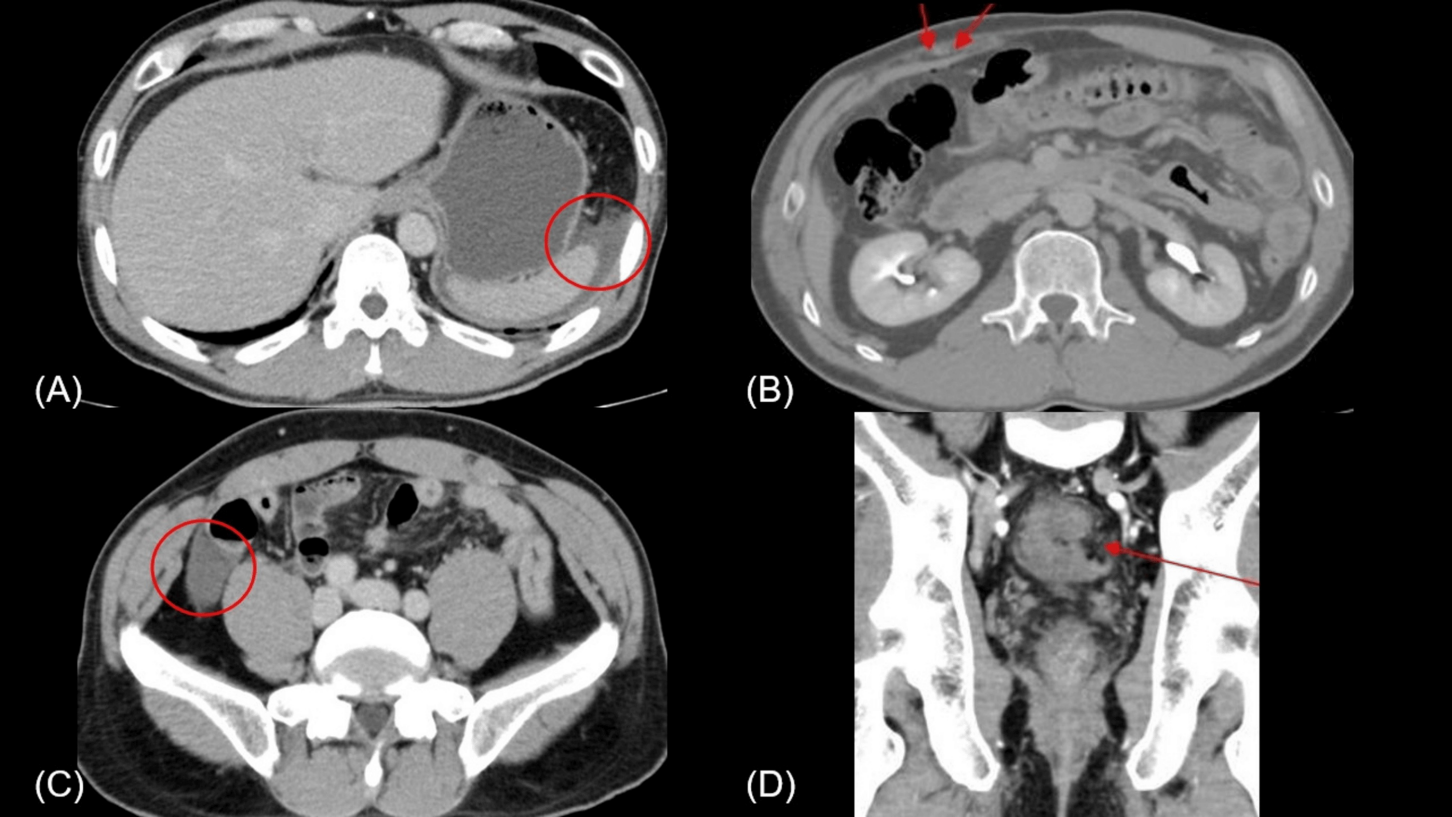
Computed tomography scans upon hospital arrival: the red circles reveal the presence of ascites surrounding the spleen (A) and ascending colon (C). The red arrows indicate scattered free air in the lower abdomen (B) and around the rectum (D).

Abdominal computed tomography (CT) revealed the presence of ascites surrounding the spleen and ascending colon, scattered free air in the lower abdomen, and poor mucosal contrast around the rectum (Figure 1). No foreign bodies were found.

**Figure 2:**
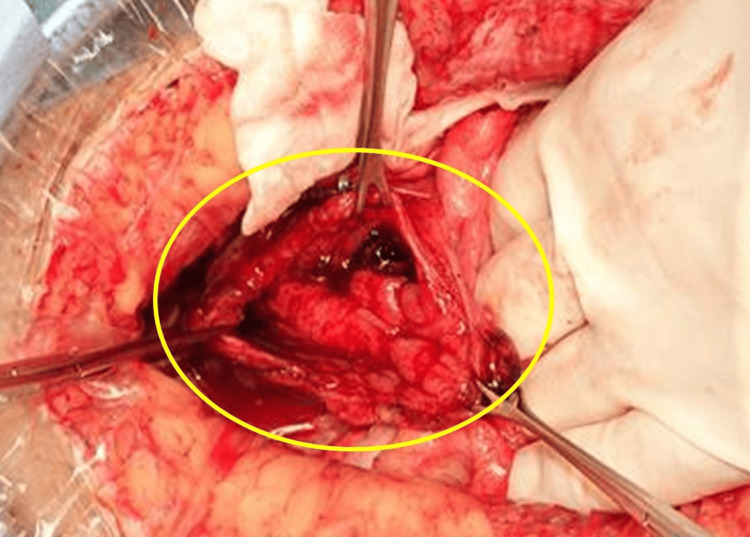
The yellow circle indicates the 10 cm full-thickness injury from Rs to Ra.

Under general anesthesia, a laparotomy was performed in the supine position through a median incision in the lower abdomen. The ascites was serous, and a 10-cm full-thickness injury from Rs to Ra area was found (Figure 2). Given the extensiveness of the injury, primary closure via transverse intestinal anastomosis using Albert-Lembert sutures alone was considered to carry a high risk of suture failure; therefore, a diverting sigmoid colostomy was performed to reduce the risk of anastomotic leakage. The patient was subsequently hospitalized for a prolonged period because of a surgical site infection, and he was discharged on day 49. An outpatient lower gastrointestinal endoscopy revealed no neoplastic lesions and indicated that the injured area was in good condition. The colostomy was closed six months later.

## Discussion

Most studies have reported rectal foreign bodies associated with sexual activity, with increasing frequency in recent years. The most commonly reported items are sexual toys (35.7%), glass bottles (17.5%), and food (11.2%) [1]. Although most cases are successfully removed transanally, if the foreign body reaches the colon, it is twice as likely to require laparotomy than if it is confined to the rectum [3]. Furthermore, depending on the type of rectal foreign body and insertion duration, it may also be removed transabdominally [2]. A laparoscopic-assisted approach may be successful [4]. Conversely, patients’ symptoms may vary considerably. In some instances, patients present with unexplained abdominal pain, and the foreign body is discovered to have been inserted after treatment. The patient’s sense of shame may impede the accuracy of the interview; thus, the presence of empathetic and considerate staff, or the application of AI technology, could be considered beneficial.

Gastrointestinal injuries caused by rectal foreign bodies are reported to be 67% AAST grade I and 33% grades II-IV. Furthermore, 64% of grade I injuries were successfully treated without surgery [5]. Conversely, most gastrointestinal perforations are attributed to mucosal damage from hard foreign bodies [6], and studies have reported delayed gastrointestinal mucosal necrosis on day 4 after insertion of a sausage [7]. However, no studies have reported cases related to condom use, making this the first such case in the world.

**Figure 3:**
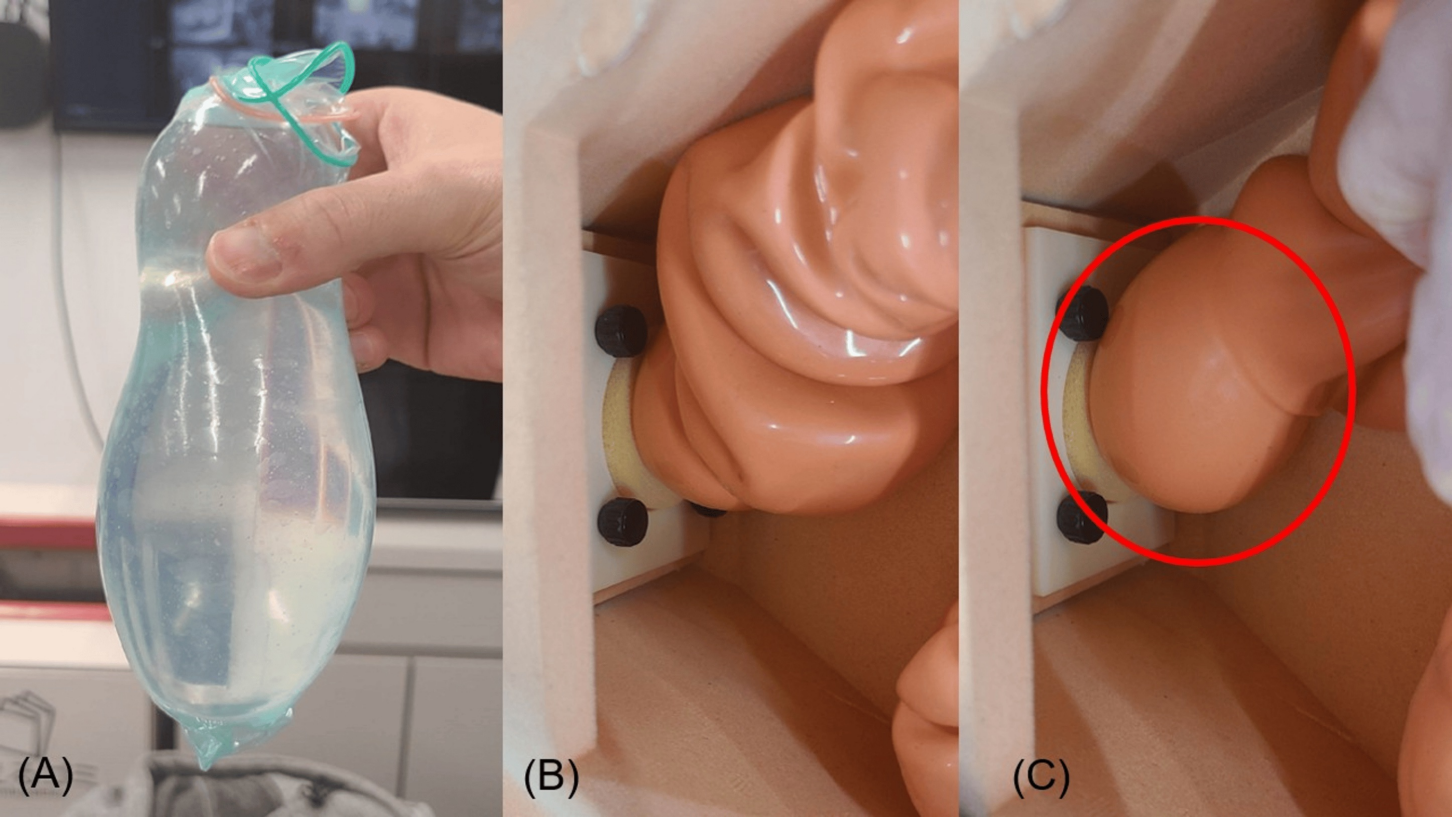
Water was injected into a triple-layered condom until the size of a hand (A). Upon insertion into the model of the lower gastrointestinal tract (B), there was a pronounced dilation on the oral side of the peritoneal reflection (C).

A comprehensive examination of a real condom and a vinyl chloride model of the lower gastrointestinal tract, and a pertinent literature review were conducted to ascertain whether the present injury mechanism truly resulted in gastrointestinal tract perforation. First, water was injected into a condom covered with two additional condoms, resulting in triple layers as reported by the patient, to create a fist-sized object. Upon insertion into the model, a pronounced dilation was observed on the oral side of the peritoneal reflection (Figure 3). It is postulated that the intestinal tract does not dilate because of the lack of available space on the anorectal side of the peritoneal inversion. However, it is hypothesized that the intestinal pressure is increased at the oral side of the inversion because sufficient space is present, which allows for intestinal dilation and facilitates the movement of water in the condom to the oral side. Furthermore, abdominal muscle exercises may have increased the intra-abdominal or intestinal pressure [8,9]. Thus, the patient’s intra-abdominal or intestinal pressure is increased by the insertion of a condom and abdominal muscle exercises. In addition, sexual arousal is achieved through the afferent pathways of the pelvic and pubic nerves. In this case, the patient asserted that he had performed the act based on the information he had obtained from the Internet.

In this case, the patient was initially approached with some skepticism because the condoms had been discarded. An emergency laparotomy was deemed necessary because of CT findings of a gastrointestinal perforation. Despite a thorough examination, no foreign body was found. However, serous ascites fluid, which was not fecal fluid, was observed, and intra-abdominal contamination was not remarkable. In contrast, the bowel injury was extensive, measuring approximately 10 cm, and the wound margins were also contused, which is consistent with condom rupture.

## Conclusions

Insertion of a water-filled condom into the anus can cause upper rectal injury under conditions of increased abdominal pressure, such as during abdominal exercises. Even in the absence of a foreign body within the rectum, the possibility of perforation should be considered. Consideration of potential feelings of shame or embarrassment is essential in patient assessment and management.
